# COVID-19 outcomes in patients with a history of immune-mediated glomerular diseases

**DOI:** 10.3389/fimmu.2023.1228457

**Published:** 2023-09-12

**Authors:** Philipp Gauckler, Jana S. Kesenheimer, Duvuru Geetha, Balazs Odler, Kathrin Eller, Timothee Laboux, Federico Alberici, Mattia Zappa, Natasha Chebotareva, Sergey Moiseev, Marco Bonilla, Kenar D. Jhaveri, Julie Oniszczuk, Vincent Audard, Denise Costa, Gianna Mastroianni-Kirsztajn, Annette Bruchfeld, Masahiro Muto, Martin Windpessl, Gert Mayer, Andreas Kronbichler

**Affiliations:** ^1^ Department of Internal Medicine IV (Nephrology and Hypertension), Medical University Innsbruck, Innsbruck, Austria; ^2^ Department of Psychology, University of Innsbruck, Innsbruck, Austria; ^3^ Division of Nephrology, Johns Hopkins University, Baltimore, MD, United States; ^4^ Department of Medicine, University of Cambridge, Cambridge, United Kingdom; ^5^ Division of Nephrology, Department of Internal Medicine, Medical University of Graz, Graz, Austria; ^6^ Nephrology Department, Univ. Lille, Centre Hospitalier Universitaire de Lille (CHU Lille), Lille, France; ^7^ Institut Pasteur de Lille, Univ. Lille, Inserm, Centre Hospitalier Universitaire de Lille (CHU Lille), Lille, France; ^8^ Division of Nephrology and Dialysis, Department of Medical and Surgical Specialties, Radiological Sciences, and Public Health, University of Brescia and Azienda Socio Sanitaria Territoriale (ASST) Spedali Civili, Brescia, Italy; ^9^ Tareev Clinic of Internal Diseases, Sechenov First Moscow State Medical University, Moscow, Russia; ^10^ Glomerular Center at Northwell Health, Division of Kidney Diseases and Hypertension, Zucker School of Medicine at Hofstra/Northwell, Northwell Health, Great Neck, NY, United States; ^11^ Department of Nephrology and Transplantation, Rare French Disease Centre “Idiopathic Nephrotic Syndrome”, Henri-Mondor/Albert-Chenevier Hospital Assistance Publique-Hôpitaux de Paris, Inserm U955, Paris-East University, Créteil, France; ^12^ Department of Nephrology, Clinical Hospital, Federal University of Pernambuco, Recife, Brazil; ^13^ Recife Medical School, Federal University of Pernambuco, Recife, Pernambuco, Brazil; ^14^ Division of Nephrology, Federal University of Sao Paulo, São Paulo, SP, Brazil; ^15^ Department of Health, Medicine and Caring Sciences, Linköpings Universitet, Linköping, Sweden; ^16^ Department of Clinical Science, Intervention and Technology, Division of Renal Medicine Karolinska Institutet, Stockholm, Sweden; ^17^ Department of Nephrology, Juntendo University Faculty of Medicine, Tokyo, Japan; ^18^ Department of Internal Medicine IV, Klinikum Wels-Grieskirchen, Wels, Austria

**Keywords:** coronavirus, risk factor, autoimmune disease, kidney disease, glomerulonephritis, immunosuppression

## Abstract

**Introduction:**

Patients with immune-mediated glomerular diseases are considered at high risk for severe COVID-19 outcomes. However, conclusive evidence for this patient population is scarce.

**Methods:**

We created a global registry and retrospectively collected clinical data of patients with COVID-19 and a previously diagnosed immune-mediated glomerular disease to characterize specific risk factors for severe COVID-19 outcomes.

**Results:**

Fifty-nine patients with a history of immune-mediated glomerular diseases were diagnosed with COVID-19 between 01.03.2020 and 31.08.2021. Over a mean follow-up period of 24.79 ± 18.89 days, ten patients (16.9%) developed acute kidney injury. Overall, 44.1% of patients were managed in an outpatient setting and therefore considered as having “non-severe” COVID-19, while 55.9% of patients had severe COVID-19 requiring hospitalization including worse outcomes. Comparing both groups, patients with severe COVID-19 were significantly older (53.55 ± 17.91 versus 39.77 ± 14.95 years, p = .003), had lower serum albumin levels at presentation (3.00 ± 0.80 g/dL versus 3.99 ± 0.68 g/dL, p = .016) and had a higher risk of developing acute kidney injury (27% versus 4%, p = .018). Male sex (p <.001) and ongoing intake of corticosteroids at presentation (p = .047) were also significantly associated with severe COVID-19 outcomes, while the overall use of ongoing immunosuppressive agents and glomerular disease remission status showed no significant association with the severity of COVID-19 (p = .430 and p = .326, respectively).

**Conclusion:**

Older age, male sex, ongoing intake of corticosteroids and lower serum albumin levels at presentation were identified as risk factors for severe COVID-19 outcomes in patients with a history of various immune-mediated glomerular diseases.

## 1 Introduction

The coronavirus disease 2019 (COVID-19) pandemic remains a challenge to physicians worldwide. Fortunately, the development of vaccines and specific treatment options, together with a likely decreasing virulence of emerging viral strains led to a shift towards a more controllable endemic situation for most individuals (1, 2).

Certain patient groups such as patients under immunosuppressive treatment are still regarded to be at risk for more severe courses of COVID-19 due to a reduced likelihood for adequate vaccine responses and impaired viral clearance (3, 4). Immunosuppressants such as B-cell depleting agents and mycophenolic acid, which are commonly used in immune-mediated glomerular diseases, are associated with impaired vaccine efficacy (5) and severe COVID-19 outcomes (6). While patients with chronic kidney disease (CKD) (7), those undergoing continuous hemodialysis (8), or kidney transplant recipients (9) are populations with an established risk for severe COVID-19 outcomes, evidence concerning patients with glomerular disease remains scarce. The IRoc-GN international registry compared outcomes of patients with established glomerular disease with hospitalized patients without glomerular disease and found in the former higher mortality rates (15% *versus* 5%) and acute kidney injury (AKI) (39% *versus* 14%) (10). A follow-up study of this cohort identified the pre-COVID-19 estimated glomerular filtration rate (eGFR) as the main risk factor for AKI, and individuals developing AKI were less likely to reach a pre-COVID-19 eGFR during short-term follow-up (11). Still, information on the ideal management of this patient population remains unknown, and a better characterization to identify patients at particular risk for severe COVID-19 is thus warranted.

The early course of the pandemic was especially threatful particularly to this vulnerable population as there was a lack of knowledge about the pathogen, disease severity and risk factors, in combination with the absence of effective therapeutic and prophylactic measures in risk populations and an overstretched healthcare system. Lessons learned from this period of the pandemic are crucial to counteract future concerns as seen after the COVID-19 pandemic in particular but also for future pandemics in general.

Consequently, the early establishment of global collaborative registries has been suggested to identify risk factors in patients with immune-mediated kidney diseases (12). To address this, a global consortium was initiated to collect COVID-19 cases during the early period of the pandemic of patients with established glomerular disease.

## 2 Materials and methods

### 2.1 Study design and participants

A global registry (COV-GN) was set up to collect clinical data of patients with COVID-19 and established or newly diagnosed glomerular disease, which presented to several nephrology departments worldwide during the early (pre-omicron) pandemic era from March 2020 until August 2021.

The criteria for inclusion in this study were i) an established diagnosis of COVID-19, either verified via viral RNA detection or because of high clinical probability, e.g. by typical symptoms and contact to a verified case and/or characteristic radiologic findings on CT scan; ii) age ≥ 18 years at the time of COVID-19, and iii) a previously established diagnosis of a immune-mediated glomerular disease. Secondary forms of glomerular diseases and glomerular diseases that clearly are not immune-mediated (e.g. diabetic kidney disease) were excluded.

Participating centers were recruited via announcements by the European Renal Association- Immunonephrology Working Group (ERA-IWG) or approached directly.

### 2.2 Data collection

Study data were collected and managed using REDCap (Research Electronic Data Capture) electronic data capture tools hosted at Medical University Innsbruck (13, 14). REDCap is a secure, web-based software platform designed to support data capture for research studies. An electronic case report form was established and transformed onto REDCap using a web-integrated project designer. Participating centers received access to an individual REDCap account. Sub-investigators screened local patient data for eligibility and entered pseudonymized patient records assigned to their respective center via their REDCap accounts. The online survey was launched on 29.06.2020 and closed on 31.03.2022.

The following data were obtained: sex, age at diagnosis of COVID-19, height, weight, race, diagnosis of established glomerular disease, remission status (complete or partial remission, no remission, relapse) and immunosuppressive regimen (induction treatment, maintenance treatment, no immunosuppression) at time of COVID-19, clinical data on admission including O2 saturation and body temperature. Laboratory data were collected on admission, peak values during follow-up and one month (3 to 5 weeks) after diagnosis of COVID-19 and included urea, creatinine, proteinuria, albuminuria, C-reactive protein, serum albumin. Clinical outcomes of COVID-19 were assessed in categories (hospitalization, non-invasive ventilation, intensive care treatment, mechanical ventilation, kidney replacement therapy due to AKI, and mortality). Further information on prior treatment of glomerular disease included name of the medication, cumulative dose, treatment duration, and the time interval from last application of immunosuppressants used.

This study was performed in line with the principles of the Declaration of Helsinki. Approval was granted by the Ethics Committee of the Medical University Innsbruck (1215/2020).

### 2.3 Statistical analyses

The clinical outcome of COVID-19 was assessed in categories. In the survey, multiple answers could be selected. For statistical analysis, outcomes were ranked by severity, increasing from lowest to highest severity. Outcomes were further classified into two groups. Patients that were managed without hospitalization (non-severe COVID-19) were compared with those that were hospitalized, treated with non-invasive ventilation, intensive care unit (ICU) care, mechanical ventilation or died (severe COVID-19), according to the most severe outcome selected. Remission status of established glomerular disease (complete remission, partial remission, no response, and relapse) as judged by the investigator on the last clinical visit before COVID-19 was assessed and dichotomized into a “remission” group (complete + partial remission) and a “no remission” group (no response + relapse). Immunosuppressive regimen at the time of COVID-19 was assessed in three categories (induction treatment, maintenance treatment, no immunosuppression) and dichotomized into an “ongoing immunosuppression” group (induction treatment + maintenance treatment) and a “no ongoing immunosuppression” group (no immunosuppression). AKI was defined as recommended by the Acute Disease Quality Initiative (ADQI) Workgroup (15), according to KDIGO criteria, as Δ serum creatinine ≥ 0.3 mg/dl from the time of admission to peak follow-up value or new start of kidney replacement treatment due to AKI. Furthermore, AKI was staged for severity according to KDIGO criteria with peak serum creatinine 1.5-1.9 times baseline for stage 1, 2.0-2.9 times baseline for stage 2 and ≥ 3.0 times baseline or increase in serum creatinine to ≥ 4.0 mg/dL or initiation of kidney replacement therapy for stage 3 (16). To test the differences between measures over time, paired t-tests were considered. Because of the rather low sample sizes, regression analyses were not used. For differences between groups, t-tests were calculated. Mann-Whitney-U-tests were used to test the differences between dichotomous variables.

## 3 Results

### 3.1 Study population and baseline characteristics

The study included 59 patients from 11 centers, covering Europe, North and South America and Asia. Patients had a previously established diagnosis of an immune-mediated glomerular disease and were diagnosed with COVID-19 between 01.03.2020 and 31.08.2021. Baseline characteristics are illustrated in **Table 1**.

**Table 1:** Baseline characteristics at the time of COVID-19 diagnosis.

Variable		*n* (% of total, n = 59)	Mean ± *SD*
Sex	Female	28 (47.5)	
	Male	31 (52.5)	
Ethnicity	Caucasian	43 (72.9)	
	African decent	9 (15.3)	
	Asian	2 (3.4)	
	Other/unknown ethnicity	4 (6.8)	
SARS-CoV-2 vaccination status	No prior vaccination	58 (98.3)	
	Incomplete vaccination*	1 (1.7)	
	Fully vaccinated*	0	
Clinical characteristics at presentation	Age (years)	59	47.48 ± 17.92
	BMI (kg/m²)	58	25.59 ± 4.28
	O2 saturation (%, ambient air)	40	93.96 ± 6.38
	Body temperature (°C)	32	37.58 ± 0.98
Glomerular disease diagnosis	Lupus nephritis	19 (32.2)	
	ANCA-associated vasculitis	11 (18.6)	
	FSGS	10 (16.9)	
	IgA nephropathy	8 (13.6)	
	Membranous nephropathy	5 (8.5)	
	Other	6 (10.2)	
Remission status on last control (before COVID-19)	Complete remission	23 (39.0)	
	Partial remission	13 (22.0)	
	No response	12 (20.3)	
	Relapse	9 (15.3)	
Immunosuppressive regimen at time of COVID-19	Induction treatment	12 (20.3)	
	Maintenance treatment	21 (35.6)	
	No immunosuppression	24 (40.7)	

BMI, body mass index; ANCA, anti-neutrophil cytoplasmic autoantibody; FSGS, focal segmental glomerulosclerosis; COVID-19, coronavirus disease 2019; Baseline data are reported as numbers of non-missing values. Frequencies are n (% of total cohort). *Fully vaccinated defined as complete primary series (at least two or three doses of any WHO EUL vaccine) according to the World Health Organization (17).

The most common diagnoses were lupus nephritis (32.2%), followed by anti-neutrophil cytoplasmic antibody (ANCA)-associated vasculitis (18.6%), focal segmental glomerulosclerosis (FSGS) (16.9%), IgA nephropathy (13.6%) and membranous nephropathy (8.5%). About two thirds (61%) of included patients were in complete or partial remission before COVID-19, while about one third (35.6%) had insufficient glomerular disease control; 55.9% were on immunosuppressive treatment (either induction or maintenance regimen) and 40.7% were without immunosuppressive treatment at presentation.

### 3.2 Kidney related outcomes

**Figure 1:**
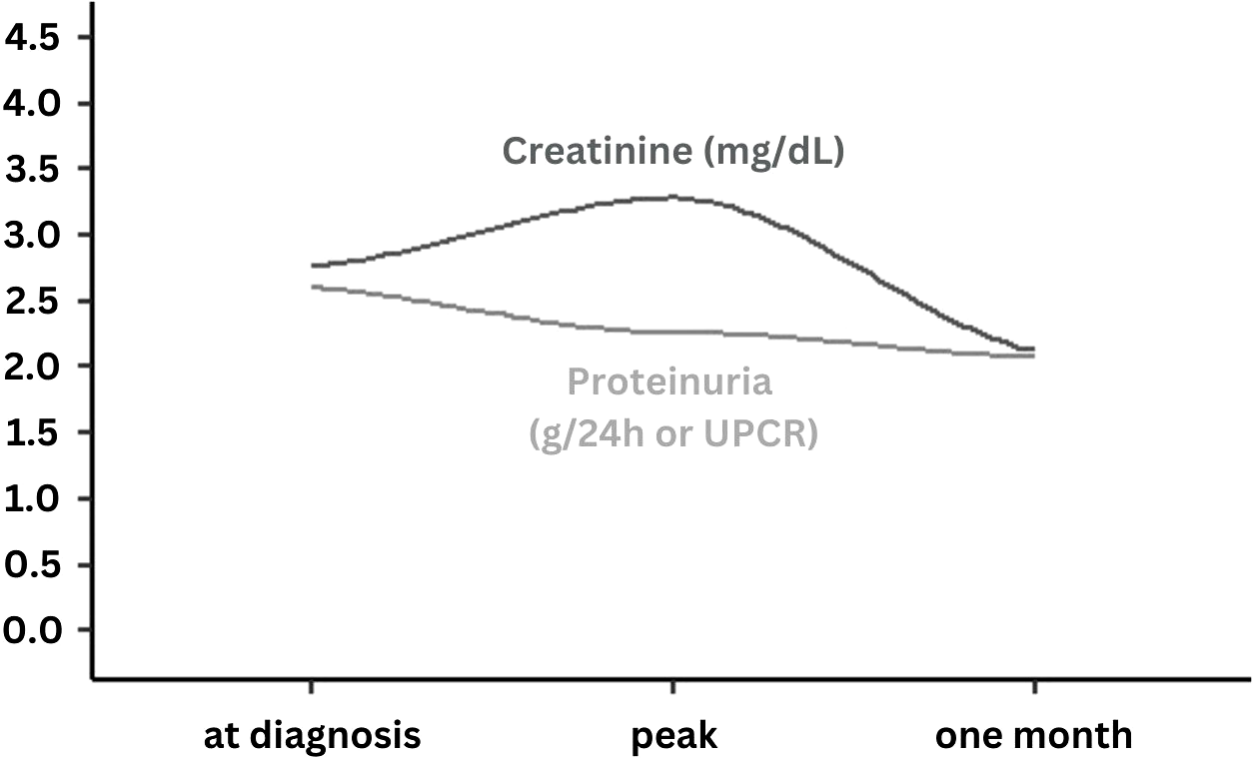
Course of serum creatinine and proteinuria levels at the time of COVID-19 diagnosis at peak and after one month follow-up. UPCR, Urine protein/creatinine ratio; Presented data are smoothed regression lines (dark grey = serum creatinine, light grey = proteinuria) according to mean values at time of COVID-19 diagnosis, at peak documented value during follow-up and at one month (3-5 weeks) of follow-up.

Mean serum creatinine and mean proteinuria at presentation were 2.76 ± 3.39 mg/dL and 2.60 ± 4.93 g/g, as assessed by spot urine measurement (urinary protein/creatinine ratio) or 24-hour urine collection (g/24h), respectively. During follow-up, serum creatinine increased to a peak mean of 3.28 ± 4.0 mg/dL (*mean_difference to baseline_* = 0.22 ± 1.03, *p* = .227), while proteinuria decreased constantly during follow-up to a minimum of 2.07 ± 3.15 mg/g (*mean_difference to baseline_* = -0.59 ± 3.31, *p* = .411). Both serum creatinine and proteinuria recovered one month after admission to a mean of 2.13 ± 1.72 mg/dL (*mean_difference to baseline_* = 0.81 ± 3.41, *p* = .176) and 2.07 ± 3.15 g/g or g/24h (*mean_difference to baseline_* = -0.10 ± 2.04, *p* = .124), respectively. Overall, ten patients (16.9%) developed AKI, of whom two patients were classified as stage I and eight patients as stage III AKI. Among those with stage III AKI, four required intermittent hemodialysis, while no continuous kidney replacement therapy or peritoneal dialysis was performed. The course of kidney-specific parameters over time is illustrated in **Figure 1**.

### 3.3 COVID-19 related outcomes

The clinical outcomes of COVID-19 ranked by severity are summarized in **Table 2**. 44.1% of patients were managed in an outpatient setting. The remaining 55.9% were hospitalized (35.6%) or even required more intensive treatment modalities, including non-invasive ventilation (3.4%), intensive care treatment (6.8%), and mechanical ventilation (5.1%). Three patients (5.1%), two of whom were hospitalized, and one outpatient died due to respiratory failure.

**Table 2:** Clinical outcomes ranked by severity.

Most severe clinical outcome	*n*	% of total sample
Classified as “non-severe”	26	44.1
No Hospitalization	26	44.1
Classified as “severe”	33	55.9
Hospitalization *(without intensive care measures)*	21	35.6
Non-invasive ventilation	2	3.4
Intensive care treatment	4	6.8
Mechanical ventilation	3	5.1
Mortality (patient deceased)	3	5.1
Total	59	100

COVID-19 outcomes ranked by severity from less severe (top) to most severe (bottom), according to the most severe outcome, if multiple outcomes were selected. Patients managed without hospitalization were classified as “non-severe”. Patients that were hospitalized or had more severe outcomes were classified as “severe”.

#### 3.3.1 Comparison of patients with severe and non-severe COVID-19 outcomes

Patients that could be managed without hospitalization (non-severe) were compared with those that were hospitalized and/or had worse outcomes (severe), as ranked in **Table 2**. Characteristics of both groups are compared in **Table 3**. Patients with non-severe versus patients with severe COVID-19 outcomes had a higher body temperature at presentation (38.50 ± 0.77°C versus 37.45 ± 0.95°C, *p* = .043), were younger (39.77 ± 14.95 years versus 53.55 ± 17.91 years, *p* = .003), and had a higher serum albumin at presentation (3.99 ± 0.68 g/dL versus 3.00 ± 0.80 g/dL, *p* = .016), respectively. Further analysis of kidney function parameters on admission (see **Supplementary Table 3**) revealed a significant association of hypoalbuminemia (serum albumin < 3.5 g/dL) with proteinuria (p = 0.040). Male sex was also significantly associated with severe COVID-19 outcomes (*p* <.001). Remission status and ongoing immunosuppression at presentation, however, were not significantly associated with severity of COVID-19 outcomes (*p* = .326 and *p* = .430, respectively).

**Table 3:** Comparison of patients with severe (hospitalized or worse, *n* = 33) and non-severe (not hospitalized, *n* = 26) COVID-19 outcomes.

		Outcome	*n*	Mean	*SD*	*p*
COVID-19 DIAGNOSIS	Body temperature (°C)	non-severe	4	38.50	0.77	.043*
		severe	28	37.45	0.95	
	O2 saturation (%, ambient air)	non-severe	11	94.91	6.25	.569
		severe	29	93.60	6.50	
	Age (years)	non-severe	26	39.77	14.95	.003**
		severe	33	53.55	17.91	
	Body mass index (kg/m²)	non-severe	26	25.52	4.68	.913
		severe	32	25.65	4.00	
	Serum urea (mg/dL)	non-severe	10	47.79	27.87	.055
		severe	29	81.14	50.23	
	Serum creatinine (mg/dL)	non-severe	13	1.77	1.25	.214
		severe	31	3.18	3.91	
	Proteinuria (g/24h or UPCR)	non-severe	10	1.83	3.20	.550
		severe	19	3.01	5.68	
	C-reactive protein (mg/dL)	non-severe	7	1.56	2.24	.145
		severe	28	7.02	9.51	
	Serum albumin (g/dL)	non-severe	5	3.99	0.68	.016*
		severe	23	3.00	0.80	
PEAK FOLLOW-UP	Serum urea (mg/dL)	non-severe	6	60.98	31.16	.203
		severe	25	101.76	74.28	
	Serum creatinine (mg/dL)	non-severe	8	1.93	1.04	.289
		severe	27	3.68	4.52	
	Proteinuria (g/24h or UPCR)	non-severe	6	1.29	2.38	.490
		severe	21	2.54	4.13	
	C-reactive protein (mg/dL)	non-severe	5	2.66	5.23	.207
		severe	23	12.25	16.16	
ONE MONTH FOLLOW UP	Serum urea (mg/dL)	non-severe	8	76.60	38.65	.540
		severe	25	65.85	43.86	
	Serum creatinine (mg/dL)	non-severe	13	1.96	1.24	.675
		severe	28	2.20	1.92	
	Proteinuria (g/24h or UPCR)	non-severe	12	2.71	3.28	.389
		severe	21	1.71	3.09	
DICHOTOMOUS*	Sex: male (*n* = 30)	non-severe	6 (20.0%)			<. 001***
		severe	25 (83.3%)			
	Sex: female (*n* = 28)	non-severe	20 (71.4%)			
		severe	8 (28.6%)			
	No ongoing immunosuppression (*n* = 24)	non-severe	12 (50.0%)			.430
		severe	12 (50.0%)			
	Ongoing immunsupression^1^ (*n* = 33)	non-severe	13 (39.4%)			
		severe	20 (60.6%)			
	Remission^2^ (*n* = 36)	non-severe	14 (38.9%)			.326
		severe	22 (61.1%)			
	No remission^3^ (*n* = 21)	non-severe	11 (52.4%)			
		severe	10 (47.6%)			
	AKI^4^ (*n* = 10)	non-severe	1 (10.0%)			.018*
		severe	9 (90.0%)			
	No AKI^4^ (*n* = 49)	non-severe	25 (51.0%)			
		severe	24 (49.0%)			
	AKI stage I (n = 2)	non-severe	0 (0.0%)			.617^#^
		severe	2 (100.0%)			
	AKI stage III (n = 8)	non-severe	1 (12.5%)			
		severe	7 (87.5%)			

UPCR, urine protein/creatinine ratio; AKI, acute kidney injury; COVID-19 outcomes were dichotomized into non-severe (= patients managed without hospitalization) or severe (= patients that were hospitalized or had more severe outcomes). ^1^defined as ongoing immunosuppressive treatment regimen (induction or maintenance treatment) at presentation; ^2^defined as complete or partial remission of established glomerular disease at presentation. ³defined as relapse or no response of established glomerular disease at presentation. ^4^defined as Δ serum creatinine ≥ 0.3 mg/dl from time of diagnosis to peak follow-up value or new start of kidney replacement treatment due to acute kidney injury. ^5^AKI staging for severity according to KDIGO criteria: peak serum creatinine 1.5-1.9 times baseline for stage 1, 2.0-2.9 times baseline for stage 2 and ≥ 3.0 times baseline or increase in serum creatinine to ≥ 4.0 mg/dL or initiation of kidney replacement therapy for stage 3; AKI stage 2 not listed (n=0). *Mann-Whitney-U-Test was used for dichotomous variables. ^#^The difference between the ratios of outcomes is statistically not significant, when comparing the groups AKI stage I and AKI stage III, p = .617 (Man-Whitney-U test).

Bold letters: * Level of significance: p < .05; ** Level of significance: p < .01; *** Level of signifiance: p < .001.

#### 3.3.2 The role of glomerular disease remission status on COVID-19 related outcomes

Patients in remission (*n* = 36, 61.0%) and without remission (*n* = 21, 35.6%) were compared (**Supplementary Table 1**). Patients without remission were significantly younger (38.48 ± 17.09 years versus 52.06 ± 15.16 years, *p* = .003) and had a lower blood oxygenation at presentation (90.04 ± 9.45% versus 95.43 ± 4.13%, *p* = .017). Serum urea and serum creatinine were significantly higher in patients without remission as compared with patients in remission at all three time points (**Supplementary Table 1**).

In terms of outcomes, the overall group differences in each category were small. Patients in remission compared with patients without remission were more often hospitalized (41.8% versus 28.6%), treated at the ICU (8.3% versus 4.8%) or received mechanical ventilation (5.6% versus 4.8%), but less frequently received non-invasive ventilation or died because of COVID-19 (both 2.8% versus 4.8%). When COVID-19 related outcomes were regarded dichotomized into no-severe or severe, as summarized in **Table 3**, patients in remission tended to have a higher risk for severe COVID-19 outcomes (61.1% versus 47.6%, respectively). However, all these differences did not reach statistical significance (**Table 3** and **Supplementary Table 1**).

#### 3.3.3 The role of prior immunosuppressive treatment on COVID-19 outcomes

**Figure 2:**
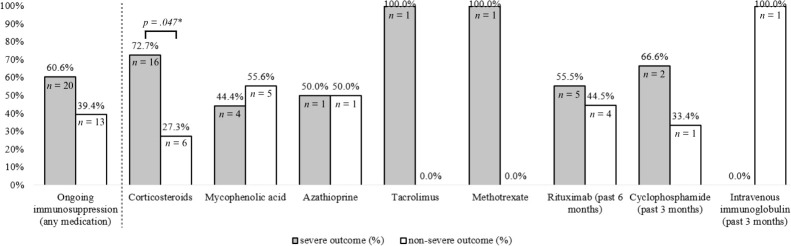
Effect of ongoing immunosuppressive medication at time of COVID-19 diagnosis on COVID-19 severity. COVID-19 outcomes were dichotomized into non-severe (= patients managed without hospitalization) or severe (=patients that were hospitalized or had more severe outcomes). Ongoing immunosuppression was defined as ongoing immunosuppressive treatment regimen (induction or maintenance treatment) at presentation. Specific medications were defined as ongoing if intake was ongoing at presentation (corticosteroids, mycophenolic acid, azathioprine, tacrolimus, methotrexate), or administered during the past 3 months (cyclophosphamide, intravenous immunoglobulin) or past 6 months (rituximab) before presentation. P-values were derived from Mann-Whitney-U-Test for dichotomous variables. *P-values were derived from Mann-Whitney-U-Test for dichotomous variables.

Patients with ongoing immunosuppressive therapy at presentation (*n* = 33, 55.9%) and without ongoing immunosuppressive therapy (*n* = 24, 40.7%) were compared, as illustrated in **Figure 2**. Patients with ongoing intake of corticosteroids at presentation were more likely to have severe (n = 16, 72.7%) versus non-severe (n = 6, 27.4%) COVID-19 outcomes (p = .047), while overall ongoing immunosuppressive treatment at presentation showed no significant association with COVID-19 outcomes (*p* = .430). No other specific immunosuppressive medication was significantly associated with COVID-19 outcomes.

#### 3.3.4 The role of glomerular disease diagnosis on COVID-19 outcomes

The distribution of the respective glomerular disease diagnoses on COVID-19 outcomes is summarized in **Supplementary Table 2**. Numerically, the risk for severe versus non-severe COVID-19 outcomes was higher for patients with ANCA-associated vasculitis (n=8 versus n=3), IgA nephropathy (n=5 versus n=3), and membranous nephropathy (n=4 versus n=1) and lower for patients with lupus nephritis (n=9 versus n=10) and FSGS (n=3 versus n=7). However, observed differences did not reach statistical significance (all p >.072)

## 4 Discussion

The COV-GN registry comprises a global cohort of patients with established immune-mediated kidney diseases, who were diagnosed with COVID-19 during an early (pre-omicron) phase of the pandemic.

After COVID-19 diagnosis, 16.9% of included patients developed AKI, while mean serum creatinine and proteinuria recovered below baseline values one month thereafter. About half of the patients had non-severe COVID-19, defined as manageable disease in an outpatient setting, while the remaining patients had severe COVID-19 with need for in-hospital treatment, including eventual intensive-care measures and/or worse outcomes. Risk factors for severe COVID-19 were older age, male sex, ongoing intake of corticosteroids and lower serum albumin levels at presentation. Remission status of glomerular disease and ongoing immunosuppressive therapy were not independently associated with severe outcomes.

### 4.1 Kidney and COVID-19 related outcomes

AKI in the context of COVID-19 is associated with high mortality and is an established risk factor for in-hospital death in patients with COVID-19 (15). The exact underlying pathophysiology of COVID-19 associated AKI is not fully understood but several mechanisms are being discussed to be involved (18). Epidemiological data on the incidence of COVID-19 associated AKI is extremely variable and depending on several factors including geographic and temporal variations during the pandemic (19), but also the lack of use of common diagnostic criteria was associated with an underestimation of AKI rates (20). Accordingly, recommendations for the diagnosis, prevention and management of COVID-19 associated AKI have been published, e.g. by the Acute Disease Quality Initiative (ADQI) Workgroup (15).

Previously, only the IRoc-GN registry reported outcomes of patients with COVID-19 and glomerular disease in comparison to patients without glomerular disease.

The AKI rate in COV-GN was substantially lower than in patients with glomerular disease reported in the IRoc-GN registry (16.9% versus 39%) and comparable with those without glomerular disease included in IRoc-GN (16.9% versus 14%) (10). This difference may be explained by the difference in reporting baseline serum creatinine values. In COV-GN, development of AKI was measured from the time of COVID-19 diagnosis, while IRoc-GN assessed baseline kidney function at a median of 3.7 (interquartile range 2.3-9.6) months before infection (11). According to a meta-analysis, the overall prevalence of AKI in patients with COVID-19 during a comparable period of the pandemic was 17% and presence of AKI was significantly associated with severe COVID-19 (21). Likewise, the mortality rate of 5.1% in COV-GN was substantially lower than a 15% mortality rate reported in IRoc-GN (10). It needs to be emphasized that varying outcomes across studies are common and may be attributed to factors such as differences in patient characteristics, methodological aspects and different biases such as selection bias (22). IRoc-GN recruited mainly patients affected during the first wave of the pandemic, which involved limited testing capacities. Thus this cohort might have been prone to more severe disease outcomes. Moreover, differing baseline characteristics of glomerular disease patients included in IRoc-GN and COV-GN need to be considered (10). Patients in IRoc-GN were older (60.3 ± 17.7 years versus 47.48 ± 17.9 years, respectively) and included fewer male patients (42.5% versus 52.5%, respectively). Also, the proportion of hospitalized patients was higher in the IRoc-GN cohort (70%), as compared to COV-GN (54%). The composition of included disease entities was broadly comparable.

### 4.2 Risk factors for severe COVID-19 related outcomes

Lower serum albumin levels at presentation were associated with severe COVID-19 outcomes in this cohort. This finding is in accordance with previous reports, indicating that hypoalbuminemia predicts severe COVID-19 outcomes in the general population (23, 24). Similarly, Waldman et al. found that serum albumin at presentation was significantly lower in patients with AKI, those requiring kidney replacement therapy and those with venous thromboembolic events, but was not associated with increased mortality in the IRoc-GN cohort (10). Other COVID-19 related outcomes were not further analyzed in IRoc-GN. In COV-GN, however, risk factors for severe COVID-19 outcomes were analyzed by stratification into two clinically relevant and similarly large groups (severe versus non-severe). This may have advantages over mortality rate as sole marker for severe COVID-19 outcomes to identify high-risk patients requiring a particularly vigilant management.

Also, in accordance with results from IRoc-GN, immunosuppressive treatment at the time of COVID-19 was not associated with adverse outcomes in both cohorts. However, and in contrast to IRoc-GN, further analysis of immunosuppressant subgroups revealed ongoing intake of corticosteroids at presentation to be associated with severe COVID-19 outcomes. These observations go in line with results from a large retrospective population-based analysis of centralized health care data from England (NHS Digital) during the second wave of COVID-19 (25). Patients with rare autoimmune rheumatic disease showed a 2.76 times risk of COVID-19-related death than the general population, with corticosteroids associated with increased risk. Overall, the discussion about risks and benefits of immunosuppressants in the context of COVID-19 is conflicting (12). Results from a large retrospective registry of patients with different rheumatic diseases showed that both, higher disease activity but also the use of rituximab and daily prednisone doses ≥ 10 mg, were associated with COVID-19-related death (26). These findings might reflect that a recent higher disease activity necessitating higher doses of immunosuppressants portends a higher risk of severe COVID-19 outcomes, as would be expected. This was also the case in a Swedish report on ANCA-vasculitis and COVID-19, which found that COVID-19 was more common in patients with higher doses of prednisone. Significant risk factors for severe COVID-19 were impaired kidney function and induction therapy whereas maintenance rituximab was not associated with severe COVID-19 disease (27).

Male sex and older age were both significantly associated with severe COVID-19 in COV-GN. Both are established risk factors for severe COVID-19 outcomes, as confirmed repeatedly throughout the pandemic (26, 28–31). Recently, sex differences in COVID-19 mortality risk have been compared among patients on kidney replacement therapy (32). In a large cohort of 3206 dialysis patients and 1204 kidney transplant recipients, male sex was a risk factor for mortality in dialysis patients but not in kidney transplant recipients. The authors assume that the use of immunosuppressants in the latter group may have narrowed sex differences and argue that the otherwise lower risk of females may be attributed to a more robust immune response to COVID-19 compared with men (33). In this cohort, immunosuppressive treatment at the time of COVID-19 did not equalize sex differences. In accordance with this, male sex was an independent risk factor for COVID-19 related death in a large cohort of 3729 patients with different rheumatic diseases, of whom 88% (3290/3729) were on immunosuppressive or immunomodulatory treatment (26).

### 4.3 Limitations

Apparent limitations of this study are its retrospective design, the relatively small cohort size, missing data at different time points and most importantly, the lack of a control group. A matched control group of patients with COVID-19 not affected by glomerular diseases would be beneficial in interpreting the results. However, even a perfectly matched control group would harbor significant risks in a retrospective study design. As an example, the indication for hospitalization will be based on other criteria in immunocompromised patients when compared with otherwise healthy individuals. Comparison with hospitalized patients only, as in IRoc-GN, provides limited advantage, particularly for the chosen outcome (severe versus non-severe COVID-19). Still, in absence of a control group, no firm conclusions on the risk of patients with glomerular diseases compared with the general population or patients with other forms of CKD is possible. Glomerular diseases are rare diseases and the establishment of the registry aimed to collect data on early evolving SARS-CoV2 strains in order to identify risk factors for severe disease courses. To recruit as many patients as possible, multiple centers worldwide were involved and inclusion criteria were defined broadly. Inclusion of patients at later time points of the pandemic would have increased the sample size, however, mass vaccination and less virulent strains would have created more heterogeneity and difficulties in interpretation of data. Although an effort was made to recruit patients globally, a relatively high proportion of patients with Caucasian ethnicity is limiting generalizability. Hospitalization of patients occurred upon discretion of the treating physician. Thus, the respective indications for hospitalization are not standardized and may vary in our cohort.

Vaccination status and currently available SARS-CoV-2-specific treatment options were initially not considered in our study. Considering the rollout of the first SARS-CoV-2 vaccines and recruitment from this time period in the respective countries, only a total of 5 patients were possibly exposed to one or more vaccination doses before presenting with COVID-19. Of these, only 1 patient received a single dose of the mRNA vaccine Spikevax^®^ (Moderna) only a few days before being diagnosed with COVID-19. No patient had a complete primary vaccination series (= fully vaccinated). Thus, the effect of SARS-CoV-2 vaccination can be neglected in the present cohort.

### 4.4 Conclusion

Our data suggest that during the pre-omicron period of the COVID-19 pandemic, patients with glomerular disease were frequently experiencing severe COVID-19 related outcomes. However, rates of severe outcomes including AKI, hospitalization and mortality were lower than in a previously published cohort of patients with glomerular disease, which may be attributed to different baseline characteristics of these cohorts and the inclusion of patients after the first wave of the pandemic, as prognosis in general improved. Of particular importance, higher age, male sex, ongoing intake of corticosteroids and lower serum albumin levels at presentation appear to be risk factors for severe COVID-19 in patients with glomerular diseases.

## Data availability statement

The raw data supporting the conclusions of this article will be made available by the authors, without undue reservation.

## Ethics statement

The studies involving humans were approved by ethics committee of the Medical University Innsbruck, Anichstraße 35, A-6020 Innsbruck, Austria. The studies were conducted in accordance with the local legislation and institutional requirements. Written informed consent for participation was not required from the participants or the participants’ legal guardians/next of kin because anonymized/pseudononymized patient data was analyzed retrospectively.

## Author contributions

AK and PG conceived the study. JK performed the statistical analysis. PG drafted the first manuscript. All authors contributed to the study design, data acquisition, data interpretation and revised and approved the final manuscript.
