# Supplementary material for: COVID-19 outcomes in patients with a history of immune-mediated glomerular diseases
Source: Front Immunol. 2023 Sep 12;14:1228457. doi: 10.3389/fimmu.2023.1228457 (PMC10520971; doi:10.3389/fimmu.2023.1228457)
Supplement: Supplementary file 1 [file DataSheet_1.pdf]

Supplementary Material

COVID-19 outcomes in patients with a history of immune-mediated glomerular diseases

Philipp Gauckler, Jana S. Kesenheimer, Duvuru Geetha, Balazs Odler, Kathrin Eller, Timothee Laboux, Federico Alberici, Mattia Zappa, Natasha Chebotareva, Sergey Moiseev, Marco Bonilla, Kenar D Jhaveri, Julie Oniszczuk, Vincent Audard, Denise Costa, Gianna Mastroianni-Kirsztajn, Annette Bruchfeld, Masahiro Muto, Martin Windpessl, Gert Mayer, Andreas Kronbichler*, on behalf of the GLOBAL-COVID-19 Glomerular Disease (COV-GN) Registry

*** Correspondence:** Andreas Kronbichler: andreas.kronbichler@i-med.ac.at

1 Supplementary material

Supplementary Table 1. Group comparison of patients with no remission (n=21) and with remission (n=36) at time of admission due to COVID-19

		Remission	*n*	Mean	*SD*	*p*
COVID-19 DIAGNOSIS	Body temperature (°C)	No	8	37.88	0.91	.356
		Yes	23	37.49	1.02	
	O2 saturation (%, ambient air)	No	11	90.04	9.45	**.017**
		Yes	28	95.43	4.13	
	Age (years)	No	21	38.48	17.09	**.003**
		Yes	36	52.06	15.16	
	Body mass index (kg/m²)	No	21	24.94	3.68	.456
		Yes	35	25.84	2.67	
	Serum urea (mg/dL)	No	12	104.17	60.30	**.003**
		Yes	26	56.70	32.38	

	Serum creatinine (mg/dL)	No	13	5.21	5.43	**.001**
		Yes	30	1.75	1.00	
	Proteinuria (g/24h or UPCR)	No	6	3.62	3.39	.604
		Yes	22	2.39	5.41	
	C-reactive protein (mg/dL)	No	10	9.56	13.04	.070
		Yes	24	3.77	5.20	
**PEAK FOLLOW-UP**	Serum urea (mg/dL)	No	11	144.25	87.07	**.001**
		Yes	20	66.16	37.17	
	Serum creatinine (mg/dL)	No	11	6.82	5.79	**<.001**
		Yes	24	1.66	0.93	
	Proteinuria (g/24h or UPCR)	No	6	4.24	4.94	.177
		Yes	20	1.79	3.42	
	C-reactive protein (mg/dL)	No	9	17.33	23.08	.104
		Yes	19	7.32	8.69	
**ONE MONTH FOLLOW UP (MFU)**	Serum urea (mg/dL)	No	12	89.42	48.41	**.029**
		Yes	21	56.48	34.10	
	Serum creatinine (mg/dL)	No	16	3.21	2.18	**.001**
		Yes	25	1.43	0.81	
	Proteinuria (g/24h or UPCR)	No	12	3.48	3.47	.051

		Yes	21	1.27	2.72	
		Yes	29	0.80	1.15	
OUTCOMES*	No hospitalization	No		11 (52.4%)		.326
		Yes		14 (38.8%)		
	Hospitalization	No		6 (28.6%)		.186
		Yes		15 (41.8%)		
	Non-invasive ventilation	No		1 (4.8%)		.852
		Yes		1 (2.8%)		
	Intensive care	No		1 (4.8%)		.458
		Yes		3 (8.3%)		
	Mechanical ventilation	No		1 (4.8%)		.614
		Yes		2 (5.6%)		
	Mortality	No		1 (4.8%)		.697
		Yes		1 (2.8%)		

UPCR, urine protein/creatinine ratio; Remission was defined as complete or partial remission of established glomerular disease at time of admission due to COVID-19. No remission was defined as relapse or no response of established glomerular disease at time of admission due to COVID-19.

**Note.* Mann-Whitney-U-Test was used for dichotomous variables.*

Supplementary Table 2. Distribution of glomerular diseases on COVID-19 outcomes.

	COVID-19 outcome		overall	*p*-value* (comparison to all diagnoses)
	Non-severe	severe		
Lupus nephritis	10 (52.6%)	9 (47.4%)	19	.365
ANCA-associated vasculitis	3 (27.2%)	8 (72.8%)	11	.217
FSGS	7 (70.0%)	3 (30.0%)	10	.072
IgA nephropathy	3 (37.5)	5 (62.5%)	8	.690
Membranous nephropathy	1 (20.0%)	4 (80.0%)	5	.261
Other	2 (33.3%)	4 (66.7%)	6	.580
Overall	26	33	59	

ANCA, anti-neutrophil cytoplasmic autoantibody; FSGS, focal segmental glomerulosclerosis; COVID-19, coronavirus disease 2019

*** Note.** *p*-value refers to a non-parametrical Mann-Whitney-Test, two-sided, alpha = .05.

Supplementary Table 3. Group comparison of hypoalbuminemia with kidney function parameters at presentation.

	Hypoalbuminemia	No hypoalbuminemia	p-value *
Proteinuria at presentation	4.12 (6.33)	0.25 (0.20)	.040*
Serum creatinine at presentation	3.55 (5.15)	1.98 (1.03)	.331
eGFR at presentation	40.86	52.82	.359

	(28.98)	(33.61)	

eGFR, estimated glomerular filtration rate

Note. *Hypoalbuminemia was defined as serum albumin level < 3.5 g/dL; eGFR was calculated using the race free CKD-EPI 2021 creatinine equation. * p-value refers to a t-Test for independent groups, two-sided, alpha = .05, taking into account the (in)equality of group variances.*
